# Intracranial Hemorrhage Due to Potential Rupture of an Arteriovenous Malformation after BNT162b2 COVID-19 mRNA Vaccination in a Young Korean Woman: Case Report

**DOI:** 10.3390/vaccines10030362

**Published:** 2022-02-25

**Authors:** Byoung Hoon Kim, Myung Chul Yoo

**Affiliations:** Department of Physical Medicine & Rehabilitation, College of Medicine, Kyung Hee University Hospital, 23 Kyung Hee Dae-ro, Dongdaemun-gu, Seoul 02447, Korea; hibyounghoon@gmail.com

**Keywords:** COVID-19 vaccines, arteriovenous malformation, intracranial hemorrhage

## Abstract

Inoculation with the Pfizer-BioNTech coronavirus infection-19 (COVID-19) vaccine (BNT162b2) has been approved in Korea. Although it is generally safe, several possible side effects have been reported. The present report describes a 28-year-old woman who developed an intracerebral hemorrhage in her right temporal lobe after the first dose of the Pfizer-BioNTech COVID-19 vaccine. The patient complained of a persistent headache for four days after the first dose, along with right third nerve palsy and drowsiness. Non-enhanced brain computed tomography confirmed a 5.0 × 3.7 × 5.0 cm^3^-sized intracranial hemorrhage in the right temporal lobe due to the rupture of an arteriovenous malformation (AVM). Transfemoral cerebral angiography revealed that blood was supplied to the AVM by the right middle cerebral artery branch and drained into the right transverse sinus. The patient underwent surgical treatment for AVM nidus removal with hematoma evacuation on the day of admission. Her condition stabilized 10 days postoperatively. These findings indicate that clinicians should be aware that cerebral hemorrhage caused by AVM rupture may be a side effect of inoculation with the BNT162b2 mRNA COVID-19 vaccine.

## 1. Introduction

Cerebral arteriovenous malformation (AVM) is an abnormal tangling of blood vessels in the brain with arteries connecting directly to veins without intervening cerebral tissue [1]. The majority of cases of AVM are asymptomatic, with most patients with AVM remaining unaware of this condition prior to manifesting neurological symptoms, such as convulsions or headache. These symptoms may be indicators of intracerebral hemorrhage due to AVM rupture, with the latter often accompanied by subarachnoid hemorrhage.

Five vaccines against SARS-CoV-2, the causative agent of coronavirus disease 2019 (COVID-19), have been approved to date by the European Medicines Agency and have been shown to reduce mortality and hospitalization rates. Two of these are messenger RNA-based vaccines encoding the spike protein of SARS-CoV-2, BNT162b2 (Pfizer-BioNTech, NY, USA) and mRNA-1273 (Moderna, Cambridge, MA, USA), whereas the other two are adenoviral vector-based vaccines encoding the spike protein, ChAdOx1 nCoV-19 (Oxford/AstraZeneca, Cambridge, UK) and Ad.26.COV2.S (Janssen, New Brunswick, NJ, USA) [2,3].

Although there is a consensus that the benefits outweigh the risks [4], rare but serious side effects have been reported. For example, the development of transverse myelitis following administration of the Oxford/AstraZeneca vaccine has raised concerns about the neurological complications of COVID-19 vaccines [5]. Other rare side effects include Guillain–Barré syndrome and thrombosis with thrombocytopenia syndrome after administration of the Janssen adenovirus-vector based COVID-19 vaccine and myocarditis and multisystem inflammatory syndrome after administration of the Pfizer-BioNTech and Moderna COVID-19 mRNA vaccines [6,7].

Therefore, the side effects associated with these vaccines are of great interest for public health and the surveillance of vaccine safety. The present report describes a 28-year-old Korean woman who developed neurological symptoms 4 days after the administration of the first dose of the Pfizer-BioNTech COVID-19 mRNA vaccine (BNT162b2) and was later found to have a right temporal intracerebral hemorrhage due to AVM rupture.

## 2. Case Presentation

A 28-year-old woman with no significant previous medical history received her first dose of the Pfizer-BioNTech COVID-19 vaccine (BNT162b2) on 9 September 2021. Four days later, she developed severe headaches and vomiting. She was admitted to the emergency room due to the sudden onset of right gaze palsy, with decreased motor function and altered mental status. At the time of admission, she was lethargic and disoriented. Her blood pressure was 130/50 mmHg, her pulse rate was 98 beats per min, her respiratory rate was 20 cycles per min, and her body temperature was 37.3 °C.

COVID-19 polymerase chain reaction tests of nasopharyngeal and oropharyngeal swabs were negative for the virus. Her initial laboratory test results are presented in Table 1. Her white blood cell count was elevated at 12,770 cells/μL, her C-reactive protein concentration was 5.21 mg/dL (reference value < 0.5 mg/dL), her fibrinogen level was 303 mg/dL (reference value 200–400 mg/dL), and her D-dimer concentration was mildly elevated, at 0.95 µg/mL (reference value < 0.5 µg/mL).

There were no specific findings of young age stroke except for a mildly elevated complement hemolysis 50 level of 108.86 U/mL; however, the results of her liver and renal function tests were unremarkable (Table 1). She was alert when she left home, but her level of consciousness deteriorated to drowsy after arriving at the emergency department. Upper motor neuron signs were present in both lower extremities. A neurological examination showed a Glasgow Coma Scale (GCS) score of 12 (M3V4E5), and she was responsive to pain, including withdrawing her arm in response to pain, but her verbal responses were inappropriate.

Her right pupil measured 6 mm and was unreactive to direct light, whereas her left pupil measured 2 mm and displayed a sluggish reaction to direct light. Evaluation of motor strength using the Medical Research Council (MRC) scale showed scores of 3/5 in her right arm and 2/5 in her left arm and in both legs. Her neurologic status continued to deteriorate, and her GCS score worsened to 9 (M2V4E3).

**Figure 1:**
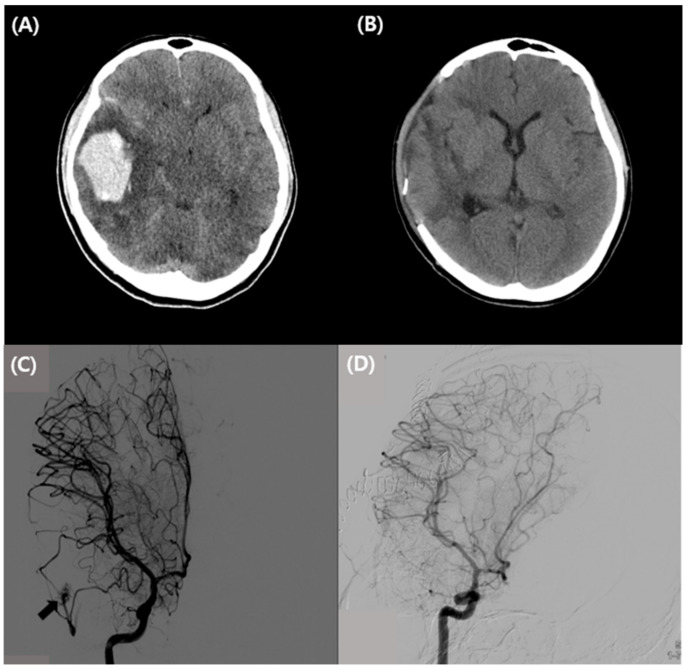
Initial and post-operative brain CT and TFCA images in the patient. (**A**) Preoperative axial image on non-enhanced brain CT, showing a 5.0 × 3.7 × 5.0 cm^3^-sized intracranial hemorrhage in the right temporal lobe with perilesional edema and mass effect with mild left side midline deviation. (**B**) Postoperative non-enhanced brain CT image 30 days after craniectomy showing marked resolution of a residual hematoma in the right cerebral hemisphere and improvements in perilesional edema and mass effect. (**C**) Preoperative TFCA showing that blood was supplied to the brain AVM by the right middle cerebral artery branch and drained into the right transverse sinus (arrow). (**D**) Post-operative TFCA 30 days after craniectomy showing removal of the AVM nidus and no residual shunt flow. Abbreviations: CT, computed tomography; TFCA, transfemoral cerebral angiography; and AVM, arteriovenous malformation.

A non-contrast brain computed tomography (CT) scan of the patient’s head showed a 5.0 × 3.7 × 5.0 cm^3^-sized intracranial hemorrhage in the right temporal lobe with perilesional edema and mass effect with mild left side midline deviation (Figure 1A), as well as a small subarachnoid hemorrhage in the right cerebral sulcus. Transfemoral cerebral angiography (TFCA) showed that blood was supplied to the AVM by the right middle cerebral artery branch and drained into the right transverse sinus (Figure 1C). On the day of admission, the patient underwent surgical treatment for AVM nidus removal with hematoma evacuation.

Postoperative TFCA showed no residual shunt flow in the AVM nidus (Figure 1D). Her condition stabilized on the tenth postoperative day, and she was transferred to the Department of Rehabilitation Medicine for rehabilitation. A follow-up brain CT about 1 month after surgery showed a markedly resolved residual hematoma and a state of permanent edema and mass effect (Figure 1B). At discharge, the patient showed significant improvements in the Berg Balance Scale, from 21 to 54; in the Korean-Mini Mental Status Examination (K-MMSE), from 24 to 30; and in the Modified Barthel index, from 45 to 83.

## 3. Discussion

This case study describes a young woman who was previously well, without prior medical history, who presented with neurological symptoms due to brain AVM rupture four days after the first dose of the Pfizer-BioNTech COVID-19 vaccine. Brain CT and TFCA confirmed a right temporal lobe hemorrhage due to AVM rupture. Laboratory tests showed non-specific increases in white blood cells, the erythrocyte sedimentation rate, and concentrations of D-dimer and C-reactive protein; however, the patient was negative or normal for cytoplasmic and perinuclear antineutrophil cytoplasmic antibodies, rheumatoid factor, cryoglobulins, antinuclear antibodies, anti-DNA, C3, C4, and immunoglobulin. Cerebral AVM is associated with a significant risk of intracerebral hemorrhage due to the direct shunting of arterial blood into the complex venous vasculature, with inflammation being a major contributor to the development of cerebral AVM [8].

To date, four vaccines have been approved by the U.S. Food and Drug Administration to prevent COVID-19 in individuals aged 16 years and older. These include two messenger RNA-based vaccines encoding the spike protein of SARS-CoV-2, BNT162b2 (Pfizer-BioNTech) and mRNA-1273 (Moderna), and two adenoviral vector-based vaccines encoding the spike protein, ChAdOx1 nCoV-19 (Oxford/AstraZeneca) and Ad.26.COV2.S (Janssen) [3].

It is generally not possible to establish a definitive causal relationship between vaccination and the development of potential side effects, particularly because the latter are uncommon. The most frequent neurological symptoms of COVID-19 include dizziness, headache, pain, muscle spasms, myalgia, and paresthesia; however, most of these side effects are relatively mild and transient [9]. Rare neurological side effects include stroke, myocarditis, Guillain–Barré syndrome, Bell’s palsy, acute disseminated encephalomyelitis, and transverse myelitis [10].

Intracerebral and/or subarachnoid hemorrhage has been observed in several individuals who have received the BNT162b2 COVID-19 mRNA vaccine [11]. The Ministry of Health, Labor and Welfare in Japan initiated inoculation of healthcare workers with this vaccine on 17 February 2021. Other vaccines have not yet been approved in Japan. To date, five individuals in Japan have died of intracerebral and/or subarachnoid hemorrhage following BNT162b2 vaccination, with four being women.

One of these women experienced nothing remarkable after the first dose of vaccine but was found dead 4 days later at home. Postmortem imaging showed a hematoma at the left cerebellopontine angle compressing the brainstem with subarachnoid hemorrhage. The incidence of death from intracerebral and/or subarachnoid hemorrhage in Japanese women who received this vaccine is disproportionately high, suggesting that neurological side effects may be associated with the COVID-19 mRNA vaccines.

The Pfizer-BioNTech COVID-19 vaccine relies on messenger RNA (mRNA). One of the proteins encoded by SARS-CoV-2 mRNA is called the spike or S protein, which is expressed on the surface of the virus. The Pfizer-BioNTech COVID-19 mRNA vaccine encodes a fragment of the S protein. Injection of the vaccine results in the expression of S protein fragments by host cells. These fragments are expressed on cell surfaces, inducing the production of specific anti-S protein antibodies. These antibodies neutralize the virus by preventing SARS-CoV-2 from attaching to host cells by inhibiting the binding of viral S protein to angiotensin-conversion enzyme 2 receptor on host cells, thereby, protecting the host against subsequent viral infections [12].

To date, SARS-CoV-2 infection has caused more than 4 million deaths worldwide, often due to excessive or abnormal host immune responses [13]. Although the benefits of vaccination outweigh the risks, vaccination is inevitably complicated by infrequent immune-mediated side effects, as proinflammatory stimuli may expose individuals to the development of maladaptive immune responses [14,15]. Vaccination may activate abnormal innate and acquired immune responses, leading to infectious cascades. The activation of systemic immune pathways in specific individuals can play a role in the development of AVM rupture [16].

SARS-CoV-2 infection has been associated with AVM ruptures caused by vascular injury and brain hemorrhage. For example, three male schoolchildren, aged 5, 6 and 14 years, presented with abrupt neurological deterioration due to AVM ruptures after contracting COVID-19, suggesting an association between viral infection and AVM ruptures [15]. Specifically, inflammatory responses to COVID-19 may alter AVM hemodynamic patterns and increase the risk of rupture. In addition, four patients experienced subarachnoid hemorrhage (SAH) associated with COVID-19 [17].

Although the precise mechanism by which virus infection causes cerebral hemorrhage in patients with pre-existing blood vessel injury remains unclear, a systemic hyperinflammatory status was shown to correlate positively with vascular injury [18]. COVID-19-induced systemic hyperinflammation has been associated with hyperviscosity [17] as well as with injuries to cerebral blood vessels resulting from collagen breakdown and increased permeability of the blood–brain barrier (BBB). COVID-19 was found to induce a cytokine storm (hypercytokinemia) with increases in the blood concentrations of mediators, such as IL-1β, IL-6, and TNF-α, thereby, enhancing systemic inflammation and leading to vascular injury [19].

To date, no studies have assessed the side effects of COVID-19 vaccine in individuals with abnormal blood vessels and blood vessel injury. Although the onset of cerebral hemorrhage following vaccination in the present patient may have been coincidental, enhanced systemic inflammation is believed to be the pathogenic mechanism directly or indirectly related to abnormal vessel injury caused by the vaccine. Direct endothelial damage induced by the COVID-19 vaccine can lead to the development of vasculitis [20,21], whereas inflammation and extracellular matrix remodeling following vaccination may result in AVM growth and rupture [22,23].

Alternatively, peptides encoded by the vaccine and expressed by the viral spike protein may show molecular mimicry with peptides expressed on host endothelial cells, particularly following non-specific adjuvant effects. Additionally, upregulation of proinflammatory cytokines can induce the overexpression of cell adhesion molecules by endothelial cells in AVM, enhancing the recruitment of leukocytes, especially neutrophils and macrophages, thereby, promoting the instability of the nidal vasculature [24,25]. Recruitment of neutrophils and macrophages to the site of injury can increase the concentrations of leukocyte-derived metalloproteinases, which can damage the vessel walls of AVMs and lead to their rupture [8].

The future management of patients who experience cerebral hemorrhage after vaccination should be similar to that for patients who experience post-vaccination thrombosis with thrombocytopenia syndrome (TTS). Guidelines have been issued by the World Health Organization (WHO) to prevent COVID-19 infection in patients with post-vaccination TTS. TTS has been reported in individuals vaccinated with the ChAdOx-1 (AstraZeneca) and Ad26.COV2-S (Johnson & Johnson) non-replicating adenovirus vector-based COVID-19 vaccines, however, to our knowledge, not yet in individuals vaccinated with the BNT162b2 (Pfizer–BioNTech) or mRNA-1273 (Moderna) mRNA vaccines.

Individuals who develop TTS following the first dose of an adenovirus-vector-based vaccine should not receive the second dose, thereby, avoiding repeated exposure to the causative antigen. Adenovirus vector-based COVID-19 and other vaccines are contraindicated in patients with a prior history of heparin-induced thrombocytopenia and those with major venous or arterial thromboses accompanied by thrombocytopenia.

Similarly, a second dose of mRNA vaccine is contraindicated in individuals with a history of hemorrhagic stroke. Upon vaccination, individuals should be informed that they may experience neurological symptoms, and that, if they do so, they should immediately visit the emergency room.

## 4. Conclusions

In summary, this report describes a young Korean woman who experienced a right temporal intracerebral hemorrhage due to AVM rupture, starting 4 days after receiving the first dose of the BNT162b2 COVID-19 mRNA vaccine. To our knowledge, this is the first report of a patient who experienced an intracerebral hemorrhage with AVM rupture after COVID-19 vaccination. Although no cause–effect relationship could be determined, the findings in this patient indicate the importance of identifying rare adverse effects of the vaccine. Clinicians should, therefore, be aware that cerebral hemorrhage caused by AVM rupture may be a side effect of the BNT162b2 COVID-19 mRNA vaccine.

## Figures and Tables

**Table 1:** Laboratory findings of the patient.

Factor	Amount	Units	Reference Value
Hemoglobin	12.3	g/dL	12–16
White blood cell count	12,770	/μL	4000–10,000
Platelet count	177,000	/μL	150,000–450,000
Erythrocyte sedimentation rate	10	mm/hr	0–20
C-reactive protein	5.21	mg/dL	<0.5
Blood urea nitrogen	12	mg/dL	8–20
Creatinine	0.43	mg/dL	0.51–0.95
Fibrinogen	303	mg/dL	200–400
D-dimer	0.95	µg/mL	<0.5
CH 50 (Complement Hemolysis)	108.86	U/mL	41.25–94.39
C3	130	mg/dL	88–201
C4	25.4	mg/dL	16–47
RA Factor	<10.5	IU/mL	<20
Fibrinogen	303	mg/dL	200–400
FDP (Fibrinogen degradation product)	<2.50	µg/mL	<5
Antithrombin III	110	%	80–120
Protein C Activity	132	%	70–130
Protein S Activity	115	%	+65–140
Anti-beta 2 GP1 IgG	1.0	G units	Normal < 20
Anti-Cardiolipin IgG	4.1	GPL U/mL	Negative < 12.0
Anti-Phospholipid IgG	2.4	U/mL	Negative < 12.0
Lupus Anticoagulant Ab	Negative		Negative
Homocysteine	6.4	µg/mL	0–15

## Data Availability

Not applicable.
